# Endoscopic Evaluation and Management of Gastrointestinal Bleeding in Patients with Ventricular Assist Devices

**DOI:** 10.1155/2012/630483

**Published:** 2012-02-28

**Authors:** Marty M. Meyer, Scott D. Young, Benjamin Sun, Maher Azzouz, Michael S. Firstenberg

**Affiliations:** ^1^Division of Gastroenterology, Hepatology, and Nutrition, The Ohio State University Medical Center, Columbus, OH 43210, USA; ^2^Department of Cardiothoracic Surgery, The Minneapolis Heart Institute, Minneapolis, MN 55407, USA; ^3^Division of Cardiac Surgery, The Ohio State University Medical Center, Columbus, OH 43212, USA

## Abstract

The optimal diagnostic approach and yield for gastrointestinal bleeding (GIB) in patients with ventricular assist devices (VAD) are unknown. We explored the etiology of bleeding and yield of upper and lower endoscopy, balloon-assisted enteroscopy, and video capsule endoscopy in the evaluation of GIB in patients with VADs. *Methods*. All VAD patients with overt gastrointestinal bleeding and drop in hematocrit from April 1, 2000 to July 31, 2008 were retrospectively reviewed. The endoscopic evaluation of each episode was recorded. Overall yield of EGD, colonoscopy, balloon-assisted, and video capsule endoscopy were evaluated. *Results*. Thirty-six bleeding episodes occurred involving 20 patients. The site of GIB was identified in 32/36 episodes (88.9%), and the etiology of bleeding was determined in 30/36 cases (83.3%). Five VAD patients underwent VCE. The VCE exams demonstrated a high yield with 80% of exams identifying the etiology of GIB. Endoscopic intervention was successful in 8/9 attempts. No adverse events were recorded. Two patients required surgical intervention for GIB. *Conclusion*. Upper, lower, video capsule, and balloon-assisted enteroscopies are safe and demonstrate a high yield in the investigation of gastrointestinal bleeding in VAD patients. Medical centers caring for VAD patients should employ a standardized protocol to optimize endoscopic evaluation and intervention.

## 1. Introduction

Ventricular assist devices (VAD) are an established therapy for patients with end-stage heart failure and have been shown to improve survival compared to optimal medical management [1, 2]. As patients with long-term devices survive longer, unique complications or unconventional presentations of common medical problems, including gastrointestinal bleeding (GIB), are becoming more frequent. Management algorithms in this challenging patient population are poorly defined. GIB is common in patients with VAD. Risk factors for bleeding are complex and insufficiently understood. These include concomitant use of anticoagulant and antiplatelet agents, chronic intestinal ischemia induced by nonpulsatile devices, and the hypothesized development of arteriovenous malformations related to suspected Von Willebrand's disease. Recent evidence suggests that these patients are at an increased risk of bleeding beyond what would typically be expected for patients requiring anti-coagulation for other indications [3].

VAD patients typically undergo a standard endoscopic evaluation in the setting of acute GIB. Limited data exists describing the yield and safety of endoscopy for the evaluation of overt gastrointestinal hemorrhage in VAD patients. In non-VAD patients, video capsule endoscopy (VCE) has become the main diagnostic tool for the evaluation of obscure gastrointestinal bleeding, which will affect approximately 5% of patients presenting with gastrointestinal hemorrhage [4]. Capsule endoscopy has been recommended as the third test offered in the evaluation of patients with GIB after upper endoscopy and colonoscopy are negative [5]. The use of VCE in the evaluation of obscure-overt GI bleeding in VAD patients is limited to isolated case reports [6–9]. We describe a cohort of VAD patients admitted for overt gastrointestinal bleeding and report the high yield of standard upper and lower endoscopy as well as VCE in the diagnostic evaluation. We also describe a high rate of success for endoscopic intervention in VAD patients. Finally, we propose new endoscopic algorithms with an emphasis on early VCE in the evaluation of this difficult-to-manage patient population.

## 2. Methods

### 2.1. Patients

 Following Institutional Review Board approval, we performed a retrospective review of all patients who underwent VAD placement at The Ohio State University Medical Center. We reviewed all hospitalizations and procedure reports to identify those patients who demonstrated any episodes of overt gastrointestinal bleeding. Between April 1, 2000 and July 31, 2008, 162 patients were implanted with 190 VADs. Of these, only those who were evaluated for symptomatic gastrointestinal bleeding as defined by hematemesis, melena, and/or hematochezia with associated decrease in baseline hematocrit were included in the data analysis.

### 2.2. Endoscopic Evaluation

Patients underwent primary evaluation with standard upper endoscopy and/or colonoscopy within 24–48 hrs upon admission to the hospital. Upper endoscopy was performed after at least a six-hour fast colonoscopy was carried out after a bowel purge consisting of 4 L polyethylene glycol (Golytely). Endoscopic procedures were performed at the bedside. Conscious sedation was administered per standard protocol with intravenous fentanyl and midazolam. Continuous telemetry, blood pressure, pulse oximetry, and VAD flow rates were monitored during the procedures. In total, 33 EGDs, 15 colonoscopies, five enteroscopies (three balloon assisted) and five VCE were performed in the evaluation of 36 episodes of bleeding.

VCE was performed using Pillcam SB (Given Imaging Ltd, Yoqneam, Israel), according to the manufacturer's instructions (Given Diagnostic Imaging System, M2A capsule; Given Imaging). Preparation for VCE included 2L polyethylene glycol (Golytely) the evening before the procedure and 80 mg simethicone immediately prior to capsule ingestion. Additional laxative was not given if capsule ingestion immediately followed a negative colonoscopy. VCE examinations were interpreted within 24 hours by one of four gastroenterologists experienced in reading VCE examinations. The videos were read at a rate of 15 frames/second. All patients tolerated their bowel preps, conscious sedation, and endoscopic procedures without hemodynamic or physiologic complications.

### 2.3. Data Collection

 Data were collected from our Institutional Mechanical Support database and the patients' electronic medical record. Variables including age, race, gender, VAD device, etiology of congestive heart failure, medical comorbidities, home medications, dates of GI bleeding and associated decrease in hemoglobin/hematocrit, coagulation parameters, blood transfusion requirement, and endoscopic diagnosis were all recorded.

## 3. Results

### 3.1. Patients

 Within the enrollment period, 20 patients (12.3%) were identified with overt GIB, totaling 36 episodes. These patients were treated with 27 devices including: Heartmate II (*n* = 6, Thoratec Corporation, Pleasanton, CA); Heartmate XVE (*n* = 4); Thoratec IVAD (*n* = 2); VentrAssist (*n* = 2, Ventracor Limited, Chatswood, NSW, Australia); Abiomed BVS500 (*n* = 6, Abiomed Corporation, Danvers, MA) Levitronix CentriMag VAS (*n* = 7, Levitronix LLC, Waltham, MA). Baseline demographics, bleeding presentation, and transfusion requirements are presented in Table 1. Thirteen patients had one episode of GIB, whereas two patients had two episodes, four patients had three episodes, and one patient had seven episodes. Three patients had a history of pre-VAD peptic ulcer disease, including one prior GIB episode. Two patients had normal pre-VAD screening colonoscopies. Fourteen patients were chronically anticoagulated with warfarin. Seventeen patients were taking aspirin and 8/17 ASA patients were taking an additional antiplatelet agent (clopidogrel, ticlopidine, or dipyridamole). Fourteen patients were treated with either a proton pump inhibitor or histamine receptor blocker.

### 3.2. Endoscopic Findings

 The site of GIB was identified in 32/36 episodes (88.9%), and the etiology of bleeding was determined in 30/36 cases (83.3%). The etiologies for these bleeding episodes are outlined in Table 2, and the yields for the endoscopic exams are summarized in Table 3. The utilization of endoscopic studies by exam type is presented in Table 4. Five VAD patients underwent VCE after a standard endoscopic evaluation failed to make a diagnosis. VCE was performed on average 3.6 days after hospital admission (range 1–7 days). The VCE exams demonstrated a yield of 80% for identifying the etiology of GIB. The fifth VCE exam, performed on hospital day 7, was limited by gastric retention. This patient had the VCE repeated with endoscopic placement into the duodenum after recurrent bleeding. VCE showed evidence of an ulcer with active bleeding in the distal duodenum. Follow-up push enteroscopy could not identify the lesion, and the patient was discharged in stable condition. The results of this positive VCE were not included in our analysis as the exam was performed outside the inclusion dates. The four positive VCE exams demonstrated two actively bleeding arteriovenous malformations in the jejunum, one bleeding cecal ulcer, and one nonbleeding submucosal jejunal mass. The cecal ulcer demonstrated a nonbleeding visible vessel and dual endoscopic therapy with epinephrine injection and hemoclip application was successful. The patients with active bleeding from AVMs underwent peroral single-balloon enteroscopy. One patient had active bleeding that was distal to the reach of the balloon enteroscope. A visceral angiogram was performed and was normal. The patient was placed on subcutaneous octreotide therapy with no further evidence for GI bleeding and was discharged with supportive care while continuing his anticoagulation. The second patient underwent balloon enteroscopy that did not demonstrate any lesions or active bleeding. Her anticoagulation was held and she was discharged home on ASA and persantine therapy without further evidence for repeat GI hemorrhage.

Overall, endoscopic intervention in the form of epinephrine injection, bipolar electrocautery, argon plasma coagulation, and hemoclip application was successful in 8/9 attempts. No adverse events were recorded as a result of the aforementioned endoscopic procedures. Two patients required surgical intervention for acute GIB. The first patient underwent a right hemicolectomy for ischemic colitis and died of refractory multiorgan failure several days postoperatively. The second patient required a gastrotomy and excision of an actively bleeding marginal ulcer that had failed endoscopic therapy. She recovered postoperatively and was discharged.

## 4. Discussion

 As a result of the increased survival after VAD implantation, GIB has demonstrated increasing relevance in the long-term management of VAD patients. These individuals typically require continuous anticoagulation and antiplatelet therapies to minimize the risk of device-associated thromboembolic complications or catastrophic pump failure. Management algorithms in this challenging patient population are poorly defined. Despite the increased rates of GIB, discontinuation of anticoagulation and/or antiplatelet agents is often contraindicated due the risk of VAD thrombosis. Our study is a comprehensive evaluation of GIB in a cohort of VAD patients. In addition, we present a novel algorithm to enhance the evaluation and yield of this challenging patient population. Several authors have published their results of successful VCE in VAD patients [6–9]. These four case reports focus on the safety of VCE use in VAD patients, whereas our series highlights the actual endoscopic yield, safety, and outcomes for various modalities investigating GI hemorrhage in this cohort.

 Video capsule endoscopy has become the main diagnostic strategy for obscure gastrointestinal hemorrhage. Small bowel follow-through radiographs demonstrate a low yield (3–20%) for pathologic findings in patients with gastrointestinal hemorrhage [10]. In addition to the low yield, diagnostic imaging results may be compromised in VAD patients due to the intra-abdominal placement of the device. The superiority of capsule endoscopy was shown in a meta-analysis of patients with obscure GI bleeding. The yield of capsule endoscopy was 56%, compared to 26% for push enteroscopy and 3% for small bowel series [11].

In our series of VAD patients with overt gastrointestinal bleeding, multiple etiologies were responsible for their presentations, the most common of which was melena. Peptic ulcer disease, jejunal angioectasias, and ischemic colitis were the most common causes identified. These patients successfully underwent 58 endoscopic procedures, including five VCE and three balloon-assisted enteroscopies. No adverse events resulted from endoscopic intervention, although two patients required surgery for ongoing hemorrhage. No hemodynamic, electronic, or mechanical abnormalities were observed with VCE at our institution. Not only did VCE demonstrate a high diagnostic yield, but there was also the opportunity for targeted endoscopic therapy. Endoscopic intervention was successful in achieving hemostasis in 8/9 attempts, and those patients with lesions identified on VCE were successfully treated without surgery.

 GI bleeding associated with VAD patients continues to emerge as an important postoperative comorbidity. Our patients demonstrated their first episode of GIB an average of 57.9 days after device implantation (range 3–272 days). This contradicts the REMATCH data where patients were followed for up to 30 months without episodes of significant GIB. Although the cause for these different observations is unknown, these patients exhibit etiologies of bleeding that are not unexpected, including peptic ulcer disease, colonic ischemia and jejunal angioectasias. Despite these straightforward endoscopic diagnoses, numerous patients present with multiple episodes of GIB even with antisecretory therapy and device optimization. Thus, many VAD patients will be expected to undergo numerous endoscopic evaluations for GIB given their ongoing risks and medical comorbidities. Of note, repeat standard upper and lower endoscopy may be warranted for future episodes of bleeding, as 20% of patients with obscure bleeding are found to have lesions within the reach of conventional upper and lower endoscopes on subsequent examinations [12].

**Figure 1:**
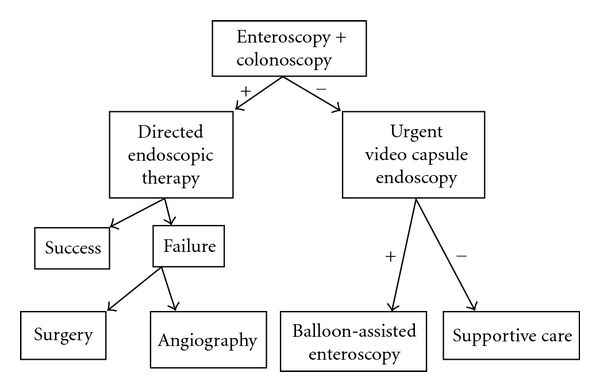
Decision algorithm for the initial evaluation of VAD patients with obscure-overt gastrointestinal bleeding.

**Figure 2:**
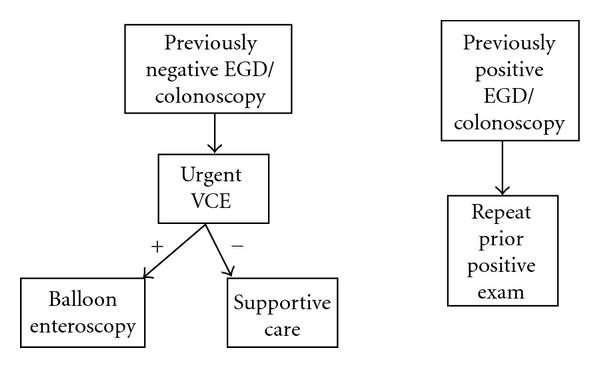
Decision algorithm for the evaluation of VAD patients with recurrent obscure-overt gastrointestinal bleeding.

 The timing of capsule endoscopy is paramount for success in finding a source of bleeding [13]. Previous investigators demonstrated a 73.3% yield for VCE in their evaluation of obscure-overt gastrointestinal bleeding, with an average time from admission to VCE of 4.1 days. Bresci and colleagues reported a higher diagnostic yield for patients with overt bleeding if the capsule study was performed within 15 days of the acute bleeding episode [14]. Our capsule studies were performed on average 3.6 days after hospital admission with a yield of 80%. When VAD patients are admitted for overt gastrointestinal bleeding, a standardized protocol should be followed. Given the prevalence of an upper digestive source of bleeding, we believe capsule studies should be performed as early as possible. The first endoscopic study should be determined by an initial versus a recurrent episode of GIB. We have developed and utilized the algorithms shown in Figures 1 and 2. The benefits of pre-VAD screening colonoscopy in determining the etiology of future GIB are unknown. However, given the future risk of GIB, with the majority of cases occurring in the proximal gastrointestinal tract, physicians should refer these patients for a pre-VAD screening colonoscopy. In addition to the benefits of colorectal cancer screening, urgent VCE may be offered earlier to those patients presenting with subsequent melena, the most common presentation among our cohort. Such an approach would maximize the yield of diagnostic endoscopy, including video capsule endoscopy, and lead to earlier targeted endoscopic therapy.

 Another important implication from prior VCE research is the long-term bleeding risk after a negative exam. Macdonald and associates reported a significant difference in rebleeding between those patients with a positive capsule examination compared to those with negative findings. Those patients with a negative study had only an 11% rate of recurrent bleeding, as compared to 42% for those patients with a positive study [15]. There is no long-term data regarding negative capsule studies in VAD patients, certainly an area for future research.

 VADs included in our study included both pulsatile and nonpulsatile devices. Several investigators have assessed the risks of axial versus pulsatile flow and the resultant risk of gastrointestinal hemorrhage. Crow et al. found a bleeding rate of 63 events/100 patient years in nonpulsatile device recipients [3]. These patients, who also receive chronic anticoagulation with warfarin, demonstrated a much higher bleeding rate than those patients with axial flow devices. Their analysis showed a rate of bleeding higher than that expected by anticoagulation alone, which was shown by other investigators to be 5.7% per year [16]. Narrow pulse pressure resulting from nonpulsatile flow has been posited to lead to the development of gastrointestinal angiodysplasia. Similar to aortic stenosis, a narrow pulse pressure may increase intraluminal pressure and dilate mucosal veins, leading to arteriovenous malformations [17, 18]. Further comparison of these two populations is difficult since the decision to use specific VADs is often based upon significant comorbidities—and in particular the risk for bleeding or a relative/absolute contraindication to anticoagulation.

 A limitation to our study includes the retrospective data collection. VAD patients may have presented to outside medical facilities for gastrointestinal hemorrhage. However, we believe this situation to be rare as our VAD patients are always transferred to us for further management. Another limitation to our study includes the combined data for pulsatile as well as nonpulsatile devices. As mentioned above, VAD selection is carefully weighed on patients' absolute or relative contraindications to aggressive anticoagulation. All patients are maintained on prophylaxis with daily aspirin. Our intent was not to ascertain the bleeding risk attributable to these differences in device type or medication regimen, but rather to assess the endoscopic yield and safety for these patients presenting with GIB. We believe these data are applicable to all VAD patients with GI bleeding as one would not alter the endoscopic workup based on VAD device type.

## 5. Conclusions

 GI bleeding is relatively common in VAD patients with varying etiologies. Traditional endoscopic procedures including EGD, colonoscopy, and push enteroscopy can be safely performed in this patient population. In addition, balloon enteroscopy and VCE are safe and valid interventions to evaluate GIB in this cohort. The early use of VCE has a high diagnostic yield in the VAD patient population. Our multidisciplinary approach has produced high yield results with an opportunity to provide early endoscopic therapy as well as minimize long-term morbidity. Given the longer survival, need for long-term anticoagulation and antiplatelet therapy, and increased incidence of GIB, we believe any medical center providing care for those with VAD should implement a standardized protocol regarding the management of gastrointestinal hemorrhage. As a result, standard and capsule endoscopy will be performed as early as possible to maximize the diagnostic yield and offer directed endoscopic therapy.

## Figures and Tables

**Table 1:** Demographic data in 20 VAD patients admitted for overt GI bleeding.

Patients (no.)	20
Total number of bleeding episodes	36
Male/female	11/9
Median age (y)	56.9 (range 41–72)
Ischemic heart disease	17
Bleeding presentation	
Melena	19
Hematemesis	9
Hematochezia	7
Hematemesis and hematochezia	1
Mean Hgb (g/dL)	9.01 (range 4.9–10.9)
Mean INR for pts on anticoagulation	2.29 (range 1.4–4.9)
Mean INR for pts not anticoagulated	1.4 (range 1.2–1.6)
Mean platelet count	215 K/ul (range 52–630)
Mean units of blood transfused*	3.3 (range 0–10)
Fresh frozen plasma*	0.75 (range 0–8)

*Per episode of bleeding.

**Table 2:** Endoscopic findings in 20 VAD patients admitted for 36 overt GI bleeding episodes.

Endoscopic findings	Number of patients (%)
Peptic ulcer disease	8 (22.2)
Small bowel angioectasias	6 (16.7)
Ischemic colitis	4 (11.1)
Erosive gastropathy	2 (5.6)
Ischemic gastritis	2 (5.6)
Herpes esophagitis	1 (2.8)
Esophageal ulcer	1 (2.8)
Colonic ulcer	1 (2.8)
Colon polyp	1 (2.8)
Bleeding site unknown	10 (27.8)
Patients with multiple etiologies of bleeding	3 (8.3)

**Table 3:** Yield of endoscopic exams.

Exam type	Positive findings (%)	Definite source of bleeding (%)
EGD	28/33 (84.8)	16/33 (48.5)
Colonoscopy	10/15 (66.7)	7/15 (46.7)
Video capsule endoscopy	4/5 (80)*	4/5 (80)
Enteroscopy	4/5 (80)	3/5 (60)
All episodes of bleeding	32/36 (88.9)	30/36 (83.3)

*The fifth VCE exam had gastric retention and was not repeated until after the study was concluded.

**Table 4:** Utilization of endoscopic studies by exam type.

Exams performed	Number of patients
EGD only	8
EGD + colonoscopy	6
Colonoscopy only	1
EGD + colonoscopy + VCE	2
EGD + VCE	1
EGD + colonoscopy + enteroscopy + VCE	2
